# Supplementary material for: Polyketide synthase gene clusters in the ericoid mycorrhizal fungus Oidiodendron maius: genome-wide prediction, expression profiling, and functional characterization of knockout mutants
Source: Front Plant Sci. 2026 Sep 16;17:1924430. doi: 10.3389/fpls.2026.1924430 (PMC13623903; doi:10.3389/fpls.2026.1924430)
Supplement: Supplementary file 1 [file SupplementaryFile1.zip › Supplementary Materials/Supplementary Folder 1.zip!/Oidma1_AssemblyScaffolds_Repeatmasked/knownclusterblast/cluster37/jgi.p_Oidma1_183251_mibig_hits.html]

| MIBiG Protein | Description | MIBiG Cluster | MiBiG Product | % ID | % Coverage | BLAST Score | E-value |
| --- | --- | --- | --- | --- | --- | --- | --- |
| AHI59116.1 | putative\_2,5-diketo-D-gluconic\_acid\_reductase | BGC0001005 | Locillomycin\_biosynthetic\_gene\_cluster | 34 | 86.4197530864 | 163 | 4.2e-40 |
| AFB35637.1 | EsmC | BGC0000935 | Esmeraldin\_biosynthetic\_gene\_cluster | 22 | 91.049382716 | 56 | 5.6e-08 |
